# Therapeutics, Its Principles and Practice

**Published:** 1889-04

**Authors:** 


Therapeutics. Its Principles and Practice. By H. C. Wood, M. D., LL.D. Seventh edition. A Work on Medical Agencies, Drugs, and Poisons, with especial reference to the Relations between Physiology and Clinical Medicine. 8vo. 1889. Philadelphia, Pa.: J. B. Lippincott Co.

These books are by the teachers of materia medica and therapeutics in three of the most prominent medical colleges. The second one is a new applicant for favor, which, if it has its due reward, it will not obtain. As a "text-book for practitioners," it is a conspicuous failure, even if practitioners needed a text-book, which is certainly doubtful, as their recitation days are usually ended when they enter the practice of a busy profession like medicine. The "State of Pharmacology" is certainly not presented in the work, in spite of Prof. Edes' declaration in the preface. This book may do very well as an epitome for students who listen to the author's lectures, saving them the labor and benefit of note-taking, but otherwise it has no claims to recognition or use at all.

Bartholow's Materia Medica is a standard, has rapidly run through six editions, and will always remain an attractive book to physicians who believe heartily in drugs, and to enthusiasts in medicine. Any one who has listened to the author's admirable lectures will find them permanently recorded in these pages, and as he turns them over, he will recall and declare to himself to be true to that statement so often heard from him, "that in properly selected cases, this remedy is undoubtedly the right thing." So is this book, and no doctor can read or study it without being benefitted. It is the work of a busy, brainy man.

Prof. Wood's "Therapeutics—its Principles and its Practice," attracts us at once. That is what we want—something practical—something usefully theoretical—something that will enable us to grow in knowledge. To be sure we are somewhat disappointed to find an old friend with a new name, and to find that his Excellency, the Honorable, is only plain John Jones, after all. But still we must acknowledge that it is a good and a great book, and to those who never saw it before, it will surely open treasures worthy of acquiring and preserving.

The introduction to this book, we have always considered the finest presentation of the scientific methods of pharmacology, and the noblest plea for higher thought and more elevated practice in medicine, that is found in medical literature. In its scope of statement, language, and breadth of ideas, it is not surpassed in medical writing, as for the book itself its numerous editions and the continued demand, speak louder than any other praise. It is a worthy production of its author, whose rank in therapeutics has and never will be questioned. No physician should be without Wood's Therapeutics. H.

